# What Does It Take to Get Somebody Back to Work after Severe Acquired Brain Injury? Service Actions within the Vocational Intervention Program (VIP 2.0)

**DOI:** 10.3390/ijerph19159548

**Published:** 2022-08-03

**Authors:** Philippa McRae, Conrad Kobel, Sue Lukersmith, Grahame Simpson

**Affiliations:** 1 Brain Injury Rehabilitation Research Group, Ingham Institute for Applied Medical Research, Sydney, NSW 2170, Australia; philippa.mcrae@yahoo.com.au; 2 Brain Injury Rehabilitation Directorate, Agency for Clinical Innovation, NSW Health, Sydney, NSW 2065, Australia; 3 Australian Health Services Research Institute, University of Wollongong, Wollongong, NSW 2500, Australia; ckobel@uow.edu.au; 4 Health Research Institute, University of Canberra, Bruce, ACT 2617, Australia; sue.lukersmith@canberra.edu.au; 5 Faculty of Medicine and Health, University of Sydney, Sydney, NSW 2006, Australia; 6 John Walsh Centre for Rehabilitation Research, The Kolling Institute, Sydney, NSW 2065, Australia

**Keywords:** traumatic brain injury, acquired brain injury, vocational rehabilitation, case management, return to work, taxonomy

## Abstract

Little is known about service actions delivered in the complex intervention of vocational rehabilitation (VR) for people with severe acquired brain injury (ABI). Scale-up of the Vocational Intervention Program (VIP) across the 12 Community teams of the NSW Brain Injury Rehabilitation Program provided an opportunity to analyse the intensity and profile of actions delivered in providing VR programs. Seventy-two participants with severe TBI were supported in returning to either pre-injury employment (FastTrack, FT, *n* = 27) or new employment (NewTrack, NT, *n* = 50), delivered by two types of VR providers (Disability Employment Service DES; private providers). VR providers documented their service actions in hours and minutes, using the Case Management Taxonomy, adapted to VR. The NT pathway required significantly higher levels of intervention in comparison to FT (25 h, five minutes vs. 35 h, 30 min, *p* = 0.048, W = 446). Case coordination was the most frequent service action overall (41.7% of total time for FT, 42.3% for NT). DES providers recorded significantly greater amounts of time undertaking engagement, assessment and planning, and emotional/motivational support actions compared to private providers. Overall duration of the programs were a median of 46 weeks (NT) and 36 weeks (FT), respectively. This study helps illuminate the profile of VR interventions for people with severe TBI.

## 1. Introduction

Acquired brain injury (ABI) includes a range of conditions including stroke, hypoxia, infections, tumours and traumatic brain injury. Within specialist brain injury rehabilitation units targeting adults of working age, the clinical profile often presents a large proportion of clients with a traumatic brain injury (TBI). This is complemented by a smaller group of people with ABI who have similar community rehabilitation needs as the TBI group.

Research among people with TBI have found that it is a global health issue and a leading cause of long-term disability and death, worldwide [1]. Return to work is a key parameter in recovery after TBI [2]. The lifetime cost of severe TBI is an estimated $4.5 million per person [3]. A major component of these costs is the lost productivity associated with poor levels of return to work (RTW) [3].

High proportions of people with TBI are employed at the time of injury, with rates ranging between 51–80% [4,5,6]. However, the motor-sensory, cognitive and behavioural changes commonly associated with moderate to severe TBI [7] create significant challenges in seeking to return to work in competitive employment settings post-injury, with rates ranging from 17–42% [4,6,8,9]. These challenges are exacerbated by the lack of access to vocational rehabilitation (VR) services and the lack of TBI-specific knowledge and skills within such services [5,10]. Many similar challenges are faced by people with other non-traumatic forms of ABI [11]. 

VR services for people with ABI can be defined as a complex community-based rehabilitation intervention [12,13,14]. A recent systematic scoping review identified five established practice models for VR in ABI [14], of which the case coordination/resource facilitation (CC/RF) model has the strongest evidence of efficacy [15]. This model involves a partnership between health-based rehabilitation and external (non-health-based) VR services in delivering RTW programs for people with ABI. However, the specific elements within this service model (e.g., assessment, education, interventions) can vary across studies, tending to be both atheoretical and pragmatic. This then leads to difficulties in finding a common language to unpack the various elements within the ‘black box’ of VR rehabilitation for this clinical group [14,16].

Work in classifying case management within the broader human services field provides a potential solution to this challenge. Case management is a care coordination strategy for the integration of care, support and services [12,17]. Providers within the Australian context typically deliver VR services for RTW employing a case management model. The complexity of case management interventions is due to the interaction of multiple interdependent and dependent components [13,18] (see Table 1). The Case Management Taxonomy (CMTaxonomy) provides a standardised approach to understand and measure the ‘what action’ of case management (the inputs and throughputs/interventions). The CMTaxonomy was developed using rigorous scoping and qualitative consensus development techniques (nominal group technique), piloted, disseminated across 11 countries, the impact analysed, published and validated [19,20,21,22,23,24,25]. For this study, the CMTaxonomy was adapted to the VR context in order to quantify the type and amount of vocational case manager interventions (actions) delivered in facilitating the return-to-work within the Vocational Intervention Program 2.0 (VIP 2.0) project.

VIP 2.0 was a 3-year implementation of new employment pathways for people with severe ABI across NSW, Australia, following an earlier proof of concept trial (VIP 1.0). The project aimed to address the low levels of RTW after TBI in NSW [9]. Significant barriers to effective RTW programs previously identified by consumers with lived experience of ABI included a lack of knowledge and understanding about ABI among the existing VR providers, linked with poor coordination between health and VR sectors [10,26]. The VIP 2.0 addressed these issues through establishing partnerships between brain injury rehabilitation services and VR providers and providing brain injury-specific resources and mentoring to the providers to build knowledge and skills in managing RTW after ABI [27,28]. The earlier controlled trial of the VIP service model (VIP 1.0) found it was effective in enabling VR providers to deliver RTW services for people with moderate to severe ABI compared to standard care [28]. The VIP 2.0 project involved the scaling up of the VIP program to a statewide level.

In seeking to quantify the type and amount of vocational case manager interventions, two key factors were thought to influence service actions, provider type and employment pathway [18]. First, providers appointed to partnerships included both government and insurance-funded service providers, with some differences between the two service models. The Australian Government Disability Employment Service (DES) program funds generalist disability providers to provide employment services for people with disability [29]. DES providers rarely employ allied health professionals to undertake assessments and guide vocational goal setting. Service provision is highly prescribed by government-set service parameters linked to payment milestones, which limits person-centred service delivery. Conversely, private VR providers funded through personal injury insurance schemes (e.g., motor vehicle, workers compensation, income protection), are resourced with allied health professionals, with greater capacity to assess strengths and restrictions related to injury. However, private providers more commonly manage physical injuries (e.g., musculoskeletal) or conditions (e.g., deafness or mental health concerns), have less exposure and therefore experience in the return to work for persons with severe injury, particularly in managing cognitive impairment [30,31].

The VIP intervention provided two RTW pathways (Fast Track [FT], return to pre-injury employer; New Track [NT], seeking new employment [32]). Previous research has found significant differences in the client profile for the two pathways, with NT clients being significantly younger, single, having more challenging behaviours and more severe injuries compared to clients undertaking FT programs [9]. Furthermore, the need for job seeking and relevant training necessary for a new position typically requires a more intensive program across a longer duration [28]. Therefore, the aims for the current study were to document and compare (i) the intensity of intervention associated with the two pathways; (ii) the profile of actions delivered in providing RTW programs, both by pathway (FT vs. NT) and VR provider (DES vs. private); and (iii) the duration of intervention by pathway and the changes in the pattern of actions delivered across time. 

## 2. Materials and Methods

### 2.1. Setting and Sample

The NSW Brain Injury Rehabilitation Program (BIRP) comprises 15 specialist brain injury rehabilitation units (12 adult and 3 paediatric) in a state-wide network providing inpatient, transitional living and long-term community-based rehabilitation services [33]. The BIRP network is part of the NSW state health department and the primary provider of specialist rehabilitation services for children and adults of working age (spanning 0–65 years) sustaining a severe TBI in NSW. A small proportion of people with ABI who fell within the same age range and had similar community rehabilitation needs as the TBI group, and were therefore part of the BIRP services were also included. 

Twenty selected VR providers (12 private, 7 DES, one DES/private) were partnered with the 12 BIRP services, amounting to 53 partnerships at the project outset, with each BIRP service having partnerships with between three and seven providers. Between December 2018 and March 2021 the 12 participating adult community rehabilitation teams identified active clients of their service with a TBI or other form of ABI with a RTW goal, to refer to the VIP 2.0. Ethics approval for the project evaluation was granted by South Western Sydney Local Health District Human Research Ethics Committee (HREC/19/LPOOL/28) with all participating clients providing informed consent. 

### 2.2. The Vocational Intervention Program

The primary focus for VIP 2.0 was to facilitate access to competitive employment. Competitive employment was defined as work in a mainstream setting, paid under an award wage. Competitive employment was differentiated from other productive activities including sheltered or supported employment, study (either tertiary, vocational or secondary), volunteerism, and home duties. 

The VIP 2.0 Providers were not co-located with the BIRP teams, though the intention was to work closely with the BIRP case managers, under an integrated care model. Integration of services commenced at the time of referral enquiry, continued with joint assessments, work placement, training, monitoring and case closure. A one-day training program was delivered at each of the 12 sites by project staff, to enable the service partners to become familiar with each other, learn about ABI and how to integrate VIP pathways within funding schemes. Training was also provided in using VIP specific tools (tailored workplace assessment tool, report forms and a web-based information exchange tool) and the data collection processes for the VIP study. A procedures manual was made available, and electronic tools stored on a password protected website. The two trainers were Occupational Therapists with substantial experience in providing VR to people with ABI. 

The FT pathway targeted people with the opportunity and some capacity to return to their pre-injury place of employment. RTW programs follow a graduated approach, tailored to the needs of each participant and employer, including strategies to manage physical and cognitive effects of injury. Participants performed suitable duties within their pre-injury position or an alternate position. The NT pathway assisted participants where there was no opportunity to resume their pre-injury employment to pursue new employment, including ‘stepping-stone’ activities of work experience, volunteer work and training courses, as well as job seeking and placement. 

Key to the VIP 2.0 was the mentoring and training resources provided to the VR providers by project staff, as many providers had little experience in providing services to people with ABI. For DES providers, the dominant groups in the Disability Employment Service (DES) system are people with physical disability (56.7%) and psychiatric disability (38.8%), and only 1.7% of the caseload comprises people with ABI [31]. Similarly, it is uncommon for private vocational providers to encounter severe brain injury. For instance, less than one per cent of work-related injuries involve head trauma (172 cases recorded in NSW in 2016–2017, from a total of 24,153 work-related injuries [31]). Typically, the DES and private providers operate independently of health/rehabilitation teams, with services often occurring sequentially, rather than concurrently. 

All VR providers delivered the two pathways (FT, NT), with funding sourced for each referred client via available funding schemes. This included insurance schemes for those with compensable injuries (motor vehicle, workers compensation and income protection) and government schemes for those without insurance coverage (the National Disability Insurance scheme and the Disability Employment Services). Participants exited the program (termed ‘case closure’) at the point they achieved their goal of sustainable employment, or sooner if issues arose that interfered with job placement or stability. Such factors included medical, social or legal issues or a downturn in business. 

### 2.3. Measure 

The CMTaxonomy for community-based case management [19,20] was selected as the tool to categorise and enable the measurement of the services provided by VIP providers. There are two taxonomy trees in the CMTaxonomy, the intervention tree (with 9 main actions, 17 actions and 8 related actions) and the service tree, which defines the level of mobility (actions performed in the community or workplace rather than case manager’s office) and the intensity of case management (frequency of actions performed).

In consultation with the taxonomy developer, some adjustments were made to the hierarchical CMTaxonomy intervention tree (case manager actions), to suit a VR context, considering the categories of both ‘parent’ and ‘child’ actions and their definitions. Only minor changes were adopted to the ‘main actions’ (parent categories) of the CMTaxonomy; with removal of ‘advising’. Further changes were made to the child category actions, with only 9 of 17 adopted for VIP (see Table 2). The CMTaxonomy glossary definitions were used for relevant actions but contextualised to the VIP program, for all ‘actions’ relevant to VIP. For example, the child category ‘’navigating’’ in CM Taxonomy is defined as “finding the most appropriate pathway through systems, services, resources and supports for the client given their context” ([20], p. 4, Appendix 2). With VIP, “navigating” is defined as “Researching and arranging the most appropriate option (including canvassing for work training, job seeking, facilitating training courses, negotiating RTW upgrades, etc.)” (see Table 2). 

The CMTaxonomy service tree was also used where the mobility (travel) and intensity (time spent with each action) were recorded by case managers.

### 2.4. Procedures

#### 2.4.1. Provider Selection

Providers were selected via an Expression of Interest process, in which 52 providers were invited to apply for VIP partnerships. Selection committees comprising multiple stakeholders selected a total of 20 providers, some appointed to a single site and others to multiple sites (ranging between two and six sites). 

#### 2.4.2. Data Collection

**Figure 1:**
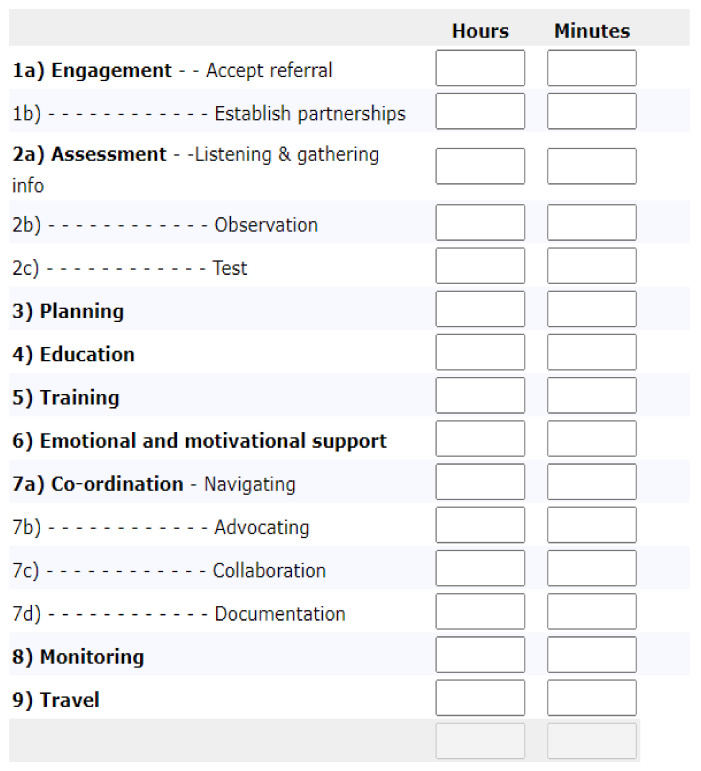
Survey form for the VIP time recording data.

A data protocol was devised to collect information on demographic, injury and employment related variables. These data were extracted from the medical files. To monitor the interventions, providers entered monthly time recording data for each client serviced into a web-based survey tool, available to providers on a password-protected website containing other program-specific resources (see Figure 1). Project staff viewed data on a monthly basis and followed up missing data and resolved data errors with providers.

#### 2.4.3. Data Analysis 

Upon database closure in April 2021 the monthly time recording data were imported to SAS software, version 9.4 for Windows, SAS Institute Inc., Cary, NC, USA, for data cleaning and analysis. The analysis included all participants who had completed the program and whose time recording was complete (*n* = 72). The time recording data were skewed to the right. Therefore, non-parametric descriptive statistics (i.e., median) and univariate statistical testing (Wilcoxon rank-sum test) were employed. It was generally expected that fewer hours would be recorded for FT compared to NT participants, and therefore one-sided tests were used. For the comparison between DES providers and private providers, two-sided tests were used. *p*-values smaller than 0.05 (*p* < 0.05) were considered statistically significant. As the analysis was descriptive in nature no correction for multiple testing was done. It should be noted that ‘percent of total’ (reported in some tables) is based on the share of total time recording (and therefore the mean) rather than the median.

Descriptive results in the ‘Service actions over time’ section are reported based on mean values as these better represent the average service actions from the providers’ perspective. The longitudinal analysis had to be limited to those participants who had completed the program, whose time recording was complete and sufficiently granular, i.e., no more than three months recorded together. Where two or three months were reported together the time recording was apportioned in equal parts to each month. Results of analysis over time are presented for all months with data for at least 10% of participants. Participants whose data had aggregated during data entry over more than three months were removed. All time recordings are reported as hh:mm (in hours and minutes).

## 3. Results

**Figure 2:**
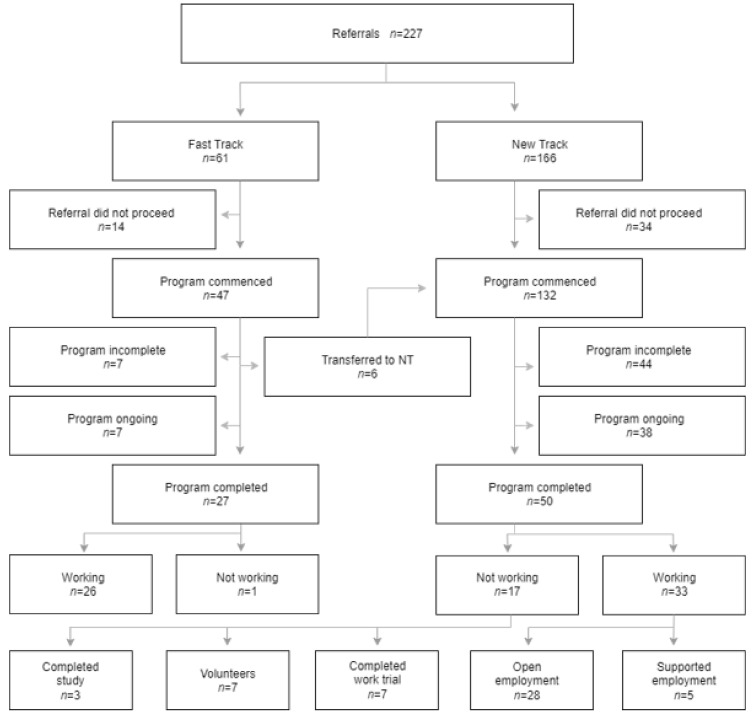
VIP 2.0 participant flow chart.

Two hundred and twenty-one people with brain injury were referred across the 12 BIRP sites to VIP 2.0 providers, with 173 individuals proceeding to program (see Figure 2). The demographic, injury vocational and psychosocial data profile for the cohort are displayed in Table 3. Comparison of the FT and NT groups found only two differences, namely that the NT group were enrolled at a significantly later time post-injury compared to the FT group, and that a lower proportion of the NT group were employed at the time of injury (see Table 3).

At the end of the project, 77 participants had completed the program (27 FT, 50 NT) and comprised the sample for this study. Among the remainder of the cohort, 51 had withdrawn (seven FT, 44 NT) and 45 individuals were still active (seven FT, 38 NT), with their programs extending beyond the project endpoint. Compared to all 77 participants who completed VIP 2.0, between-groups analysis found that the clients (*n* = 51) who withdrew from the program had commenced at a significantly later time-point post injury (median 21.0 vs. 10.0 months); were more likely to have been in rural versus metropolitan locations (76.5% vs. 54.6%); and more likely to have had extremely severe (Post Traumatic Amnesia > 28 days) injuries (51.7% vs. 34.6%, respectively).

### 3.1. Outcomes

Employment outcomes at case completion for the sample (*n* = 77) are displayed in Figure 2. For FT, 26/27 (96.30%) were working (all in open employment), with one (3.70%) not working (volunteering). For NT, 33/50 (66.0%) were working, and 17 (34.0%) not working. Of those working, 28 were in open employment and five in supported employment. Of those not working, seven were volunteering, three completed study, and seven had completed a work trial. 

### 3.2. Service Intensity: Fast Track vs. New Track

Service intensity was measured by the number of hours of service delivered. Of the 77 individuals who completed the program, a total of 72 had time recording data which were complete. This was the case for 25/27 participants who completed FT and 47/50 participants who completed the NT program. Comparing the two service pathways, the median total hours of service was 25:05 h (ranging between 3:00 to 95:49 h) in FT, which was significantly less (*p* = 0.048) than the average of 35:30 h (ranging between 2:30 to 134:00 h) in NT. 

### 3.3. Service Action Comparison: Fast Track vs. New Track 

Breaking down the results by parent categories (see Table 4), the highest hours in FT were coordination (41.7% of total time), followed by monitoring (13.5%), assessment (11.9%) and travel (11.1%). Least time was devoted to education (1.0%) and training (0.4%). Time for coordination was primarily collaboration and documentation (Table 4).

In the NT program, the highest number of hours was also recorded in coordination (42.3%), primarily collaboration and documentation. Five categories (assessment, plan ing, training, monitoring and travel) had between eight and 10% of total time recorded. The least time was spent on education (2.4%). 

Between-groups analyses found that clients in NT recorded significantly more training compared to FT (see Table 4). While the median training scores for both groups were zero, training took up only 0.4% of service time for FT clients, but 9.2% of service time for NT clients. This reflects the need to support people to learn new jobs in NT, but not required for those resuming their previous, familiar roles. 

### 3.4. Service Action Comparison: DES vs. Private Providers

Of the 72 individuals who completed the program and whose time recording data were complete, one was removed because they had changed provider type. There were 27 participants who received services from a DES provider and 44 participants from a private provider. The median total hours of service was 36:20 h (ranging between 3:00 to 134:00 h) for DES providers, exceeding the median of 29:05 h (ranging between 2:30 to 121:30 h) for private providers, however that difference was not statistically significantly (*p* = 0.169).

DES providers recorded significantly greater amounts of time undertaking engagement, assessment and planning compared to private providers (see Table 5). The greatest significant difference (*p* < 0.001) was in the level of emotional and motivational support recorded by DES providers (median 3:15, ranging between 0:00 to 20:00 h) compared to private providers (median 0:00 h, ranging between 0:00 to 17:30 h). This comprised 9.9% of DES providers’ time, compared to only 3.9% of private providers’ time. A trend was observed for private providers recording higher levels of travel time (median 2:45 h, ranging between 0:00 to 24:00 h) compared to DES providers (median 1:20 h, ranging between 0:00 to 12:05 h) but the difference was not statistically significantly (*p* = 0.069). 

### 3.5. Duration 

The overall program duration for NT participants was 46 weeks (median) and 36 weeks for FT. 

### 3.6. Service Actions by Pathway over Time

Service actions over time were evaluated for both FT and NT clients, respectively. Starting with FT, there was 18 participants whose data was sufficiently granular (i.e., no more than three months recorded together) to enable the analysis. 

Service intensity was greatest in the first two months (4:47 h on average). Thereafter the average total time recorded was relatively stable between 2:15 h and 3:33 h. For half of the participants the program ended within eight months after commencement and for three participants the program continued for up to 14 months. Thereafter the number of participants reduced further and latter months were excluded from analysis.

**Figure 3:**
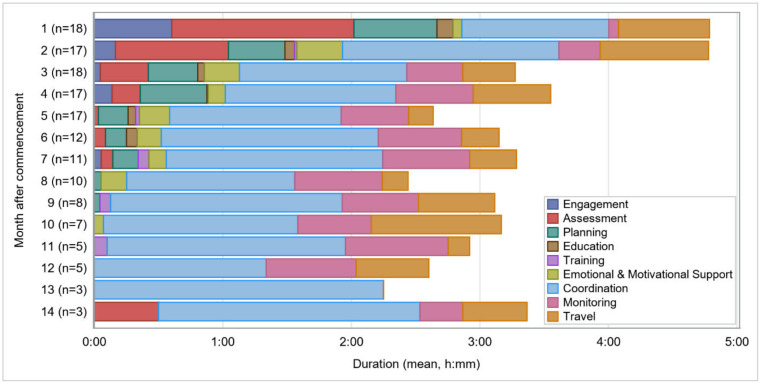
Service actions over time–Fast Track.

Figure 3 profiles the variation in service actions over time. Initially, assessment, planning and engagement account for 1:25, 0:36 and 0:39 h, respectively (see Figure 3). Their combined duration declined to 0:48 in the third month and between month six and nine their duration reduced to almost zero. With the exception of the first month, coordination was proportionately the highest category, accounting for between 1:09 h and 2:15 h per month. Monitoring and travel time recordings were relatively stable amounting to 0:30 h and 0:27 h per month, respectively.

For 40 of the 50 participants who completed the NT program, sufficient granularity allowed analysis of the service actions over time. Greatest service intensity was recorded in the second month, at 7:37, thereafter the average total time progressively declined until the six month mark. With the exception of the tenth month (5:09 h) total time recordings were relatively stable between 2:34 h and 4:00 h until month 17. During months 18 and 19 around 6:00 h were recorded before monthly service actions reduced again. For half of the participants the program ended within nine months after commencement and for five participants the program continued for up to 22 months. Thereafter the number of participants reduced further and latter months were excluded from analysis.

**Figure 4:**
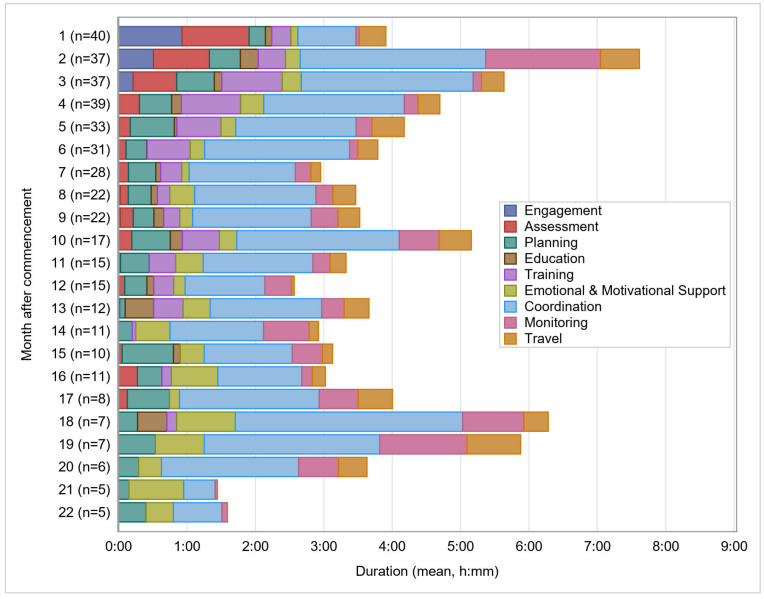
Service actions over time—New Track.

As expected, categories of assessment and engagement were initially the highest, accounting for 0:59 h and 0:56 h, respectively (refer to Figure 4). After four months their duration reduced to almost zero. Around 0:24 h every month were in planning, which was relatively stable. Training time was greatest between the second and sixth month ranging between 0:38 h and 0:53 h per month. Thereafter training time reduced. Similar to FT, co-ordination is the dominant service action over time for NT. Unlike FT, time for emotional and motivational support in NT increased over time and planning also featured across the duration of the program (having dropped to zero in the second half of the FT pathway). 

## 4. Discussion

This study has sought to unpack the ‘black box’ of the complex intervention of VR for people with severe ABI. Using the novel CMTaxonomy, the study provided one of the most detailed breakdowns of the service actions published to date. The study found that greater service intensity and duration was required for a new employment pathway, compared to a return to pre-injury employment. Additionally, the findings demonstrated that the profile of service actions between the two pathways differed. New ground has been broken in characterising the service actions as delivered by external (non-health) VR providers. Furthermore, the CMTaxonomy was able to detect differences between models of service delivery (DES vs. insurance-funded). Finally, to the best of our knowledge, no previous report has documented how the mix of service actions varies across the duration of a RTW intervention. 

As previous research has identified, a common problem in the ‘black box’ of case management and other complex interventions is defining and describing the dependent and independent components, measuring them for analysis and quality appraisal [14,18,25,34]. The CMTaxonomy was developed as a tool to support analysis of case management, adopting the definition of ‘action’ as consistent with the international ontological framework of the World Health Organization International Classification of Health Interventions (ICHI) [35]. Murray et al. [14] identified nine process steps and components in their systematic scoping review. Seven of these are aligned to the ICHI definition of an action, whilst other components align with the ICHI definition of target, or means. The CMTaxonomy (Table 2) mapped onto seven of the nine process steps ‘components’ of VR provision identified in ABI. proposed by Murray et al. [14]. The seven service components were intake, information gathering, assessment, stakeholder engagement, goal setting, intervention, and evaluation/review. In addition, the CMTaxonomy actions used for this study included education, training, emotional/motivational support and coordination (and CMTaxonomy coordination child categories of navigating, advocating, collaboration, documentation). 

Finding comparable intensity data was complicated by the differences in national or state service systems, and this was compounded by variations associated with different service models (e.g., supported employment model vs. case coordination model). Some studies only provided cost data without the underlying hours/services, and the current results could not be compared to such studies [36,37,38,39]. However, a review by Tyerman (2012) identified a number of early studies that did report intensity by hours [40]. The level of intensity was similar to an earlier Australian study, reporting findings from a single centre located within a large national government VR provider network with specialist ABI expertise [41]. O’Brien (2007) reported an average of 36 h one-to-one service within a RTW program, comparable to the VIP 2.0 (median 25 h FT, 35 h NT) [41]. The VIP 2.0 was more intensive than the original trial of the US-based Resource Facilitation model (median 8 h) which sought to provide advocacy and coordination support to people with ABI accessing state-based generic VR services [42]. On the other hand, VIP 2.0 was less intensive than the 60 h reported at the San Diego-based Work Re-entry Program at Sharp Memorial Rehabilitation Center [43]. This study was the only the second to breakdown hours by employment pathway, also finding that return to pre-injury employment (41 h) took less intervention than seeking new employment options (60 h). Finally, VIP 2.0 was less intensive than the US supported employment model, which is based on providing 1:1 on-site levels of support [37]. The supported employment model required an average of 291 h of job coaching in total, with an initial 245 h over 18 weeks to achieve ‘job stabilization’. It must be acknowledged however, that this group were more than 10 years post-injury on average, and this could, in part, account for the intensity needed. 

In terms of service actions, case co-ordination was the most frequent action across both pathways, accounting for almost half of the intervention overall (FT, 41.7%; NT, 42.3%). This finding is unsurprising, particularly when the complexity of VR for people with ABI is considered. Care coordination is a key strategy of integrated care and makes a critical contribution to person-centred care [12,44,45]. As VR is community based and the goal is for the person to return to productive roles, it requires both collaboration and coordination across health, employment, education and training, transport and social support sectors. 

The current study was also able to document two different service models (DES and private providers) that both partnered with the brain injury rehabilitation services in delivering the VIP program. The DES providers provided more engagement, assessment and planning, which was an unexpected finding, given the observation of less resources within DES providers for health/functional assessments. This may reflect the greater administrative processes for commencing a DES program. DES providers also provided more emotional/motivation support, which may relate to the positioning of the DES within the Australian social security system, such that providers attend to the need for broader social services and payments. Private providers recorded greater travel time (although this last difference only trended towards statistical significance), reflecting their more mobile operation within the community; whereas DES providers require clients to attend their offices for appointments. 

To the best of the author’s knowledge, Radford and colleagues (2018, p. 80) have been the only other study to give a breakdown of intervention components (face to face 28%; non face to face, 32%; administration, 16%, and travel 24%). Generally, these globally defined components were difficult to map onto the CMTaxonomy parent categories. The difference in travel however, the one component that could be mapped onto the current report, was substantial (5.2% DES providers, 12.8% private providers vs. 24% in Radford et al. [38]). 

The overall duration of the VIP intervention (FT median 36 weeks, NT median 46 weeks) was comparable to both UK and US studies, highlighting the lengthy duration of VR interventions for RTW (also note that a sizeable number of NT participants, N = 38, continued their engagement with providers beyond the project timeline). In the UK, VR interventions took between 32 weeks to a year [38,46]. In the US, length of VR varied across models, with program duration typically ranging from several months up to 2 years [37,47,48,49]. The current study found that over the duration of the interventions, there was also a changing profile of the mix of service actions. Across both VIP 2.0 pathways, there was a reduction in the array of service actions over time. This was most prominent in the FT pathway, in which 8/9 service actions were provided in the initial three months, reducing to 3/9 actions (co-ordination, monitoring and travel) as clients were in employment. 

## 5. Conclusions

The study results have both clinical and research implications. One of the challenges in researching VR for moderate to severe ABI, is the multiplicity of service models that have been developed to address this challenging area of practice. The CMTaxonomy has demonstrated sensitivity in characterising differences between the two service models (DES versus private) in the current study. Therefore, it may have broader utility in providing a standardised approach to characterising VR interventions delivered through a range of different service models, which could strengthen the capacity for larger scale research and evaluation of the efficacy of VR within the field. Apart from research, the value of the taxonomy in identifying the profile of different actions, either by clinical pathway (FT vs. NT) or by provider, could provide a useful framework for targeting future training in delivering VR to people with moderate to severe ABI. 

There are a number of study limitations to take into account. First, VIP 2.0 was aimed at all eligible BIRP patients in NSW, Australia within a 28-month period (which had been extended due to the COVID pandemic) and the sample size had not been powered to test specific hypotheses. The COVID pandemic may also have had an impact on the study outcomes. Second, the study was reliant on the accuracy of the data entered by the providers, and there may be some discrepancy between categories individual providers used to classify their service actions. In addition, there was variation in the timeliness with which providers entered the data, with some adding multiple months of data at the one time, rather than entering the service data on a regular weekly or fortnightly basis. This may have increased the risk of recall bias. Furthermore, we did not have data on the qualifications and skills of the providers and this may have been a factor influencing service delivery patterns. Lastly, the comparisons between subgroups such as provider types and pathways show the differences (and similarities) among those. These were done using univariate statistical testing without correction for multiple testing. Furthermore, with the number of observations available no additional analysis was undertaken to further examine the multidimensionality of the data. 

We propose that future studies should be sufficiently powered to test the hypotheses generated during this observational study.

Looking to future research, it would be important to examine the costs of the intervention. In the current study, clients were being supported through a range of different funding systems, which all applied different costing regimes, and it was beyond the scope of this study to collect such fine grained data. Next, it was not possible within the current study to undertake analyses to identify potential interactions between the clinical pathway (FT vs. NT) and provider type (DES versus private) due to the modest sample size, so a larger scale study would provide such an opportunity. In addition, future research could include measures to investigate the relative person-centredness of the two service models. Person-centred refers to the co-production of care, shared knowledge and decision making between the person and service provider. It would be possible to examine the extent to which the VR providers delivered person-centred care, characterized by an approach that is inclusive and respectful, meaningfully engages the person in their own care, assesses personal factors such as quality of life and planning involves tailored solutions focused on their strengths and participation. 

## Figures and Tables

**Table 1:** The interdependent and dependent components of case management [18,20].

Circumstance	Components Description and Examples
Whom	focus or target of the case management action—which may be the person with the health condition, e.g., brain injury OR the employer OR workplace supervisor OR the training provider
Why	purpose of the case management service, e.g., to return to work in the same job, OR to return to work in a different job OR engage in purposeful occupation
When	timing and frequency in relation to the person and employer need
What context	person’s OR training provider OR workplace context
How (service)	theoretical underpinnings/model, e.g., strengths based, managed/administrative, broker model
Where	office OR workplace based OR community based
How (skills)	Case manager qualifications and skills (e.g., health or social care or work health and safety)
Whom (sector)	Across a range of health services, social services, education providers, transport, employment related and workplace based services
What action	the case manager interventions, what they do/the actions

**Table 2:** CMTaxonomy adapted for Vocational Rehabilitation.

Main Category	Sub-Category	Description
Engagement	Accept Referral	Time spent matching the client to consultant and funding system to commence servicing.
	Establish Partnerships	Connecting with the client, family and others to establish a relationship, and develop partnerships.
Assessment	Listening and Gathering Information	Initial assessment and gathering background information from the client and relevant others (incl. verbal and written reports from BIRP case managers s, employers).
	Observation	Watching to acquire information about the work environment and/or client’s functioning.
	Test	Evaluating the client’s health using an assessment instrument (e.g., functioning assessment, vocational assessment).
Planning	-	Setting goals, priorities, actions and responsibilities with the client, employer, BIRP case managers s, funding coordinator and relevant others.
Education	-	Providing information to the client and employer to improve understanding.
Training	-	Teaching or developing the client’s skills (workplace, interview skills, work behaviours, etc.)
Emotional and Motivational Support	-	Supporting the client’s employment through vocational counselling and encouragement.
Co-Ordination	Navigating	Researching and arranging the most appropriate option (incl. canvassing for work trainings, job seeking, facilitating training courses, negotiating RTW upgrades, etc.)
	Advocating	Supporting the client in negotiations.
	Collaboration	Consulting, providing feedback and working with other service providers (incl. BIRPs, insurers, care agencies).
	Documentation	Recording notes and report writing.
Monitoring	-	Continuous acquisition of information to monitor progress (with client, employer, other parties. Includes phone, email, face to face, worksite).
Travel	-	All travel related to servicing the client.

**Table 3:** Demographic, injury, psychosocial and employment details for clients commencing VIP 2.0.

Variables	Total Sample (*n* = 173)	Fast Track (*n* = 47)	New Track (*n* = 126)	Test
Sex (*n*, %)				
Male	125 (72.25%)	33 (70.21%)	92 (73.02%)	Χ^2^ = 0.13 *p* = 0.71
Female	48 (27.75%)	14 (29.79%)	34 (26.98%)	
Age at Program Start (Years)				
M (SD)	38.50 (13.87)	39.45 (14.02)	38.15 (13.86)	*t* = −0.55 *p* = 0.59
Med (IQR)	37 (24.5)	38 (26)	36 (24.25)	
Range	18–67	18–67	18–67	
Time Post-Injury (Months)				
M (SD)	33.79 (54.62)	11.02 (14.94)	42.28 (61.28)	*U* = 1286.50 *p* < 0.0001
Med (IQR)	13 (25)	8 (7)	18.5 (34.25)	
Range	2–309	2–103	3–309	
Marital Status (*n*, %)	(*n* = 171)	(*n* = 46)	(*n* = 125)	
Married/De Facto	69 (40.35%)	23 (50.00%)	46 (36.80%)	Χ^2^ = 2.67 *p* = 0.26
Single	90 (52.63%)	21 (45.65%)	69 (55.20%)	
Separated/Divorced	12 (7.02%)	2 (4.35%)	10 (8.00%)	
Years of Pre-Injury Education	(*n* = 117)	(*n* = 26)	(*n* = 91)	
M (SD)	12.80 (2.50)	12.88 (2.67)	12.78 (2.46)	*t* = −0.19 *p* = 0.85
Med (IQR)	12 (4)	12 (6)	12 (4)	
Range	8–19	9–18	8–19	
Highest Education Achieved	(*n* = 154)	(*n* = 45)	(*n* = 109)	
Year 10 or less	50 (32.47%)	17 (37.78%)	33 (30.28%)	Χ^2^ = 0.86 *p* = 0.84
Year 12	31 (20.13%)	8 (17.78%)	23 (21.10%)	
TAFE	41 (26.62%)	11 (24.44%)	30 (27.52%)	
University	32 (20.78%)	9 (20.00%)	23 (21.10%)	
Employed at Injury (*n*, %)				
Employed	133 (76.88%)	46 (97.87%)	87 (69.05%)	Χ^2^ = 16.00 *p* < 0.0001
Not Employed	40 (23.12%)	1 (2.13%)	39 (30.95%)	
Location				
Metropolitan *	70 (40.46%)	23 (48.94%)	47 (37.30%)	Χ^2^ = 1.92 *p* = 0.17
Rural/Regional	103 (59.54%)	24 (51.06%)	79 (62.70%)	
Funding Source **				
iCare	63 (36.42%)	18 (38.30%)	45 (35.71%)	Χ^2^ = 4.82 *p* = 0.31
Other Insurance	10 (5.78%)	5 (10.64%)	5 (3.97%)	
NDIS	12 (6.94%)	3 (6.38%)	9 (7.14%)	
DES	61 (35.26%)	17 (36.17%)	44 (34.92%)	
Project Funding	27 (15.60%)	4 (8.51%)	23 (18.25%)	
Type of Injury (*n*, %)				
TBI	125 (72.25%)	37 (78.72%)	88 (69.84%)	Χ^2^ = 1.35 *p* = 0.25
ABI	48 (27.75%)	10 (21.28%)	38 (30.16%)	
PTA Duration (Days) (TBI Only)	(*n* = 108)	(*n* = 34)	(*n* = 74)	
M (SD)	35.63 (39.83)	34.94 (46.73)	35.95 (36.58)	*U* = 1058.00 *p* = 0.19
Med (IQR)	22 (31)	14.5 (38)	26 (30)	
Range	<1–183	1–183	<1–183	
TBI Severity (PTA) (*n*, %)	(*n* = 111)	(*n* = 34)	(*n* = 77)	
Mild/Moderate (<1 day)	5 (4.50%)	2 (5.88%)	3 (3.90%)	Χ^2^ = 3.42 *p* = 0.33
Severe (1–7 days)	14 (12.60%)	7 (20.59%)	7 (9.09%)	
Very Severe (8–28 days)	44 (39.64%)	13 (38.24%)	31 (40.26%)	
Extremely Severe (>28 days)	48 (43.24%)	12 (35.29%)	36 (46.75%)	

* Metropolitan BIRP sites include: Liverpool, Ryde, and Westmead. Rural/Regional BIRP sites include: Dubbo, Hunter, Illawarra, Mid-North Coast, New England, Northern, Southern, and South-Western. ** iCare includes: iCare Lifetime Care, iCare Lifetime Care (Worker’s Care), and iCare Worker’s Insurance categories. Other insurance includes: CTP and Other Insurance categories. *n* = 9 had a secondary funding source: FT—*n* = 1 DES; NT—*n* = 6 DES, *n* = 2 NDIS, *n* = 1 Project Funding. Note. *n* = 6 FT clients who transferred to NT after program commencement were counted in the FT group.

**Table 4:** Service actions by pathway.

Category	Fast Track (*n* = 25)				New Track (*n* = 47)				One Sided Wilcoxon Rank-Sum Test	
	Median	Min	Max	% of Total	Median	Min	Max	% of Total	W	*p*-Value
Engagement	01:12	00:00	02:50	3.7	01:05	00:00	06:30	3.7	545.0	0.309
Assessment	03:00	00:00	12:20	11.9	02:30	00:00	30:15	8.4	654.0	0.786
Planning	01:15	00:00	12:40	9.4	02:00	00:00	17:00	9.1	573.0	0.434
Education	00:00	00:00	02:00	1.0	00:00	00:00	06:00	2.4	457.0	0.045
Training	00:00	00:00	02:05	0.4	00:00	00:00	70:00	9.2	361.0	0.001
Emotional and Motivational Support	01:00	00:00	09:30	7.3	00:18	00:00	20:00	6.1	617.0	0.644
Coordination	09:54	00:00	50:10	41.7	14:24	00:25	78:00	42.3	461.0	0.068
Monitoring	03:55	00:00	12:30	13.5	01:24	00:00	60:00	10.0	740.0	0.965
Travel	02:10	00:00	12:15	11.1	02:00	00:00	24:00	8.7	634.0	0.712
**Total**	**25:05**	**03:00**	**95:49**		**35:30**	**02:30**	**134:00**		**446.0**	**0.048**

**Table 5:** Service actions by provider type.

Category	DES Providers (*n* = 27)				Private Providers (*n* = 44)				Two Sided Wilcoxon Rank-Sum Test	
	Median	Min	Max	% of Total	Median	Min	Max	% of Total	W	*p*-Value
Engagement	01:30	00:00	06:30	4.8	00:50	00:00	03:00	2.8	826.0	0.006
Assessment	04:15	00:00	30:15	12.3	02:27	00:00	06:24	7.1	761.5	0.048
Planning	04:30	00:00	17:00	12.0	01:00	00:00	15:48	7.2	846.5	0.003
Education	00:15	00:00	04:00	2.1	00:00	00:00	06:00	2.0	678.0	0.275
Training	00:00	00:00	70:00	8.8	00:00	00:00	30:30	5.4	708.0	0.115
Emotional and Motivational Support	03:15	00:00	20:00	9.9	00:00	00:00	17:30	3.9	903.0	<0.001
Coordination	10:05	00:00	78:00	35.5	13:25	01:42	63:42	46.5	551.5	0.619
Monitoring	03:30	00:00	22:00	9.3	01:45	00:00	60:00	12.3	679.0	0.315
Travel	01:20	00:00	12:05	5.2	02:45	00:00	24:00	12.8	440.5	0.069
**Total**	**36:20**	**03:00**	**134:00**		**29:05**	**02:30**	**121:30**		**710.5**	**0.169**

## Data Availability

Data cannot be shared publicly because of ethical issues regarding consent and potential breaches of confidentiality.
